# Voluntary wheel running access produces opposite effects in male and female rats on both palatable diet consumption and associated ventral striatal opioid- and dopamine-related gene expression

**DOI:** 10.3389/fnint.2024.1426219

**Published:** 2024-07-26

**Authors:** Courtney G. Kocum, Yonca Cam, Dusti A. Shay, Tim A. Schweizer, Ella R. Konrad, Tabitha K. Houska, Carlos A. Sardina, Todd R. Schachtman, Victoria J. Vieira-Potter, Matthew J. Will

**Affiliations:** ^1^Department of Psychological Sciences, University of Missouri, Columbia, MO, United States; ^2^Department of Nutrition and Exercise Physiology, University of Missouri, Columbia, MO, United States; ^3^Department of Biological Sciences, University of Missouri, Columbia, MO, United States; ^4^Department of Philosophy, University of Missouri, Columbia, MO, United States

**Keywords:** physical activity, sex differences, nucleus accumbens, dopamine, opioids, high fat diet, diet preferences, voluntary wheel running

## Abstract

The relationship between physical activity levels and feeding behaviors has been a focus of preclinical research for decades, yet this interaction has only recently been explored for potential sex differences. The aim of the present study was to isolate sex-dependent effects of voluntary wheel running (RUN) vs. sedentary locked wheel (SED) home cage conditions on palatability-driven feeding behavior using a 2-diet choice task between standard chow and a high-fat diet. The sex-dependent effects of physical activity on feeding behavior were examined following a within-subject novel reversal design of physical activity conditions (i.e., RUN > SED > RUN), to assess temporal sensitivity of the interaction. Following the final 2 weeks of reestablished and sustained RUN vs. SED conditions in separate groups of both males and females, reward-related opioid and dopamine gene expression within the nucleus accumbens (Acb) brain region were analyzed. Results demonstrated that the initial RUN > SED transition led to sex-dependent effects of SED condition, as males increased, and females decreased their high fat consumption, compared to their respective high fat consumption during previous RUN condition phase. Following reintroduction to the RUN condition, males decreased, and females increased their high fat consumption, compared to their separate SED control group. Last, sex-dependent shifts in ventral striatal opioid- and dopamine-related gene expression were observed to parallel the behavioral effects. The major findings of the study reveal that SED and RUN home cage conditions shift palatability-driven feeding in the opposite direction for males and females, these effects are sensitive to reversal, and these sex-dependent feeding behaviors track sex-dependent changes to critical reward-related gene expression patterns in the Acb. Considering the present high rates of sedentary behavior and obesity, furthering our understanding of the interaction between physical activity (or lack thereof) and feeding behavior should be a priority, especially in the context of these divergent sex-dependent outcomes.

## 1 Introduction

Obesity and associated diseases rank as the 2nd leading cause of preventable death in the United States (Hurt et al., 2011) and yet the prevalence rate continues to climb, with recent reports showing that 41.9% of US adults are considered obese (CDC, 2022). While obesity is most commonly attributed to the overconsumption of high calorie energy dense foods, the lack of physical activity is another major contributor. Indeed, only an estimated 24% of adults meet the US Department of Health and Human Services minimum physical activity levels (Elgaddal et al., 2022), an alarming statistic considering physical inactivity is one of the primary underlying causes of chronic disease (Booth et al., 2012; Bhaskaran et al., 2014). Therefore, two of the greatest threats to public health in the United States today are obesity and physical inactivity.

While overconsumption of high-calorie palatable food and physical inactivity may not appear related based on etiology or phenomenology, they are mediated by similar overlapping neural pathways (Kelley et al., 2005). Indeed, the neural system most clearly implicated in subserving the rewarding properties of both drugs of abuse and natural rewards is the nucleus accumbens (Acb) and its associated corticostriatal circuitry (Robbins and Everitt, 1996; Schultz et al., 1997; Kelley et al., 2005; Morales and Berridge, 2020). Considering the obesity epidemic and the associated increased risk for numerous diseases are largely attributed to an energy imbalance, it is crucial to understand and characterize the behavior and underlying physiology occurring between intake of energy dense food and physical activity levels.

Few human studies have examined the relationship between exercise and dietary patterns, and fewer have specifically investigated differences between males and females (Després et al., 1984; Anderson et al., 2001; Donnelly and Smith, 2005; Elder and Roberts, 2008; Donnelly et al., 2014; Williams et al., 2015; Beaulieu et al., 2020; Feraco et al., 2024). While some studies have shown sex differences, the interpretation is difficult given limited control over variables such as the amount and intensity of exercise and the quantity of food consumed, as well as a lack of adequately powered trials of sufficient duration. To better understand the complex interaction between physical activity and feeding behavior, preclinical animal models have employed varying methodologies, including forced treadmill or voluntary wheel running, variations in timing of wheel and diet access, diet choice composition, and number of diets made available (Miller et al., 1994; Eckel and Moore, 2004; Scarpace et al., 2010, 2012; Shapiro et al., 2011; Moody et al., 2015; Lee et al., 2017, 2019a). These methodological differences have also led to varying conclusions on the nature of this interaction of physical activity and feeding, potentially representing different underlying phenomena being modeled, including but not limited to metabolic (Cortright et al., 1997), aversion (Moody et al., 2015; Yang and Liang, 2018), or altered incentive motivational properties (Lee et al., 2017, 2019b).

One of the more recent and impactful methodological shifts toward investigating this interaction of physical activity and feeding behavior has been to examine outcomes in both male and female animals. Indeed, the vast majority of animal studies exploring this topic have only included males, yet it has long been observed that female rats exhibit greater preference and consumption of palatable diets (Pankevich and Bale, 2008) and exhibit significantly higher voluntary wheel running levels (Eikelboom and Mills, 1988; Jones et al., 1990). Recent studies examining interactions of physical activity and feeding behavior have revealed robust sex differences of physical activity on feeding behavior, with male rats showing a decrease in palatable high fat diet intake when given running access, while the same does not occur in female rats (Moody et al., 2015; Lee et al., 2017). Lee et al. (2017) revealed a differential impact of voluntary wheel running access among male and female rats’ palatable diet preferences, showing a sex-dependent shift in macronutrient diet choices. Indeed, while females with running wheel access increased their high-fat diet intake compared to sedentary females, males with running wheel access decreased high-fat intake in favor of a high-sucrose diet when compared to sedentary males (Lee et al., 2017). Last, the influence of physical activity on palatable feeding behavior in females is unaffected by ovariectomy (Moody et al., 2015), suggesting the observed sex differences are mediated by factors other than sex hormones.

Palatability-driven feeding and voluntary running behavior are both altered by endogenous opioid receptor agonists and antagonists, through systemic (Marks-Kaufman, 1982; Gosnell et al., 1990; Sisti and Lewis, 2001; Ruegsegger et al., 2016) and/or direct administration into the Acb (Zhang et al., 1998; Will et al., 2006, 2007; Ruegsegger et al., 2015). Also, both voluntary wheel running and palatable diet intake are rewarding and produce transcriptional changes to opioid-related factors in the Acb (Werme et al., 2002; Kelley et al., 2003; Greenwood et al., 2011). Although these shared mechanisms have been shown to underlie both palatability-driven feeding and voluntary wheel running, there are few studies that have investigated the interaction of these behaviors and underlying physiological processes, especially through direct comparison of both male and females. Lee et al. (2017) revealed that voluntary wheel running wheel access, compared to sedentary conditions, increased opioid-related mRNA expression in females, but not in males. The lack of opioid gene expression changes in males could have been due to the decreased high-fat diet intake observed during the wheel access, compared to sedentary condition. However, rats in this study had access to multiple highly palatable diets, so interpretation of the effects of physical activity on palatability-driven feeding specifically was limited.

The present study examined both male and female rats to examine the interaction of voluntary wheel running (RUN) and locked wheel sedentary (SED) conditions and feeding patterns using a 2-diet choice test (high fat diet and standard chow). The current study was also designed to test if RUN effects are sensitive to reversal by SED condition. Lastly, all rats were assessed for opioid- and dopamine-related gene expression changes within the ventral striatum following two-weeks of continual RUN and SED conditions.

## 2 Materials and methods

### 2.1 Subjects and housing

Sixteen male and sixteen female outbred Wistar rats (Charles River Breeding, Raleigh, NC, USA) of approximately 6 weeks of age arrived in the laboratory and were initially pair housed in separate male and female rooms. Animals remained in a climate-controlled room at a temperature of 22°C and a 12:12 h light cycle (lights on at 0700). Animal usage and procedures were approved by University of Missouri Animal Care Use Committee.

### 2.2 Experimental procedure and intervention

At approximately 10 weeks of age, rats were individually housed in standard Plexiglas cages with Tecniplast stainless steel running wheels (34 cm diameter). Running distance and duration was measured daily using Sigma Sport bicycle pedometers. Following a 1-week acclimation to voluntary wheel running access (RUN) with *ad libitum* standard chow diet (Phase 0), rats were given ab libitum access to both high fat diet and standard chow diet (for details see below) for the 4-week testing period (Weeks 2–5). Both diets were made concurrently available in separate divided sections of the cage lid, counterbalancing location within each treatment group. Daily diet consumption and body weight were recorded throughout the 4-week diet-choice period. All groups were given 1 week in the RUN condition (Phase 1) followed by 1 week of a locked wheel condition (SED) (Phase 2), to determine diet preference change between the RUN and SED conditions. During Phase 3 (weeks 4 and 5), one half of the males and females were placed back in the RUN condition (group RS-R), while the other half remained in the SED condition (group RS-S) to examine the impact of RUN and SED conditions on diet preference for a prolonged period. Animals were assigned to phase 3 cohort on day 1 of the experiment. The abbreviations RS-R and RS-S stand for “RUN-SED-RUN” and “RUN-SED-SED” respectively, in reference to which conditions the group had during phases 1, 2, and 3. At the end of week 5, three hours before onset of dark cycle (1,600 h), rats were anesthetized with CO2 (100% CO2 was introduced at the rate of 10–20% of the chamber volume per minute, regulated by a flow meter attached to the CO2 cylinder) and underwent rapid decapitation. Brains were rapidly removed, frozen and stored in −80°C for later dissection in cryostat.

### 2.3 Diet compositions

Upon arrival, rats were provided with *ad libitum* access to water and standard chow (Lab Diet 5008– 3.5 kcal/gram). After 1 week of running wheel access with standard chow, rats were given the choice between standard chow and a high-fat diet (Research Diet D12492: 5.24 kcal/gram) consisting of 60% fat, 20% carbohydrate and 20% protein. Consumption of each diet was recorded daily and standardized to kilocalories per 100 g body weight.

### 2.4 Ventral striatum RNA extraction and cDNA synthesis

Brain sections visibly identified as the ventral striatum, (Bregma 2.2–0.7 mm) (Paxinos and Watson, 1998) were sliced coronally with a cryostat (Leica BioSystems). Tissue was collected in 3 mm diameter punch samples at −20°C and then stored at −80°C until processing. Samples were lysed in Trizol using a high-speed shaking apparatus for 3 min at 25 Hz (Tissuelyser LT, Qiagen, Valencia, CA, USA) with RNase-free stainless-steel beads. RNA was separated according to the manufacturer’s instructions (TRIzol, Invitrogen, Carlsbad, CA, USA). A Nanodrop 1000 (Thermo Scientific) was used to quantify the RNA. Quality of RNA was confirmed on a 1% agarose gel. RNA was reverse transcribed using the High-Capacity cDNA Reverse Transcription kit (Applied Biosystems, Carlsbad, CA, USA).

### 2.5 Ventral striatum mRNA expression quantification

The cDNA for each sample was assayed in duplicate for target genes using SYBR Green Mastermix (Applied Biosystems Carlsbad, CA, USA). The mRNA was assessed for dopamine receptor type 1 (DRD1), dopamine receptor type 2 (DRD2), μ-opioid receptor 1 (OPRM1), proenkephalin (PENK), and Cyclophilin A. The mRNA expression values are presented as 2ΔCt, whereby ΔCTt = Cyclophilin A Ct-gene of interest Ct, and were normalized to MALE RS-S condition values. The fold changes were calculated from standard error of 2ΔCt values.

### 2.6 Statistical analysis

Analytical procedures were performed using SPSS version 29.0.1.0 (171). All values are presented as mean ± SEM. Significance for all analyses was set with an alpha value of 0.05. Rats were assigned to one of two treatment groups (i.e., RS-S or RS-R) on Day 1 of the experiment [in two cohorts (*n* = 16/cohort)] and all analyses treated these as two distinct groups from Phase 0 to Phase 3. Outcome measures for between-group and within-group comparisons were analyzed using a two-way analysis of variance (ANOVA) [Sex (MALE vs. FEMALE), group (RS-S vs. RS-R)], or across weeks/physical activity condition using a repeated measures ANOVA [Week/physical activity, sex (MALE vs. FEMALE), and group (RS-S vs. RS-R)]. Significant effects were followed by either a Tukey *post hoc* comparison or planned *post hoc* comparison *t*-tests.

## 3 Results

### 3.1 Body weight and running distance

#### 3.1.1 Body weight

**FIGURE 1:**
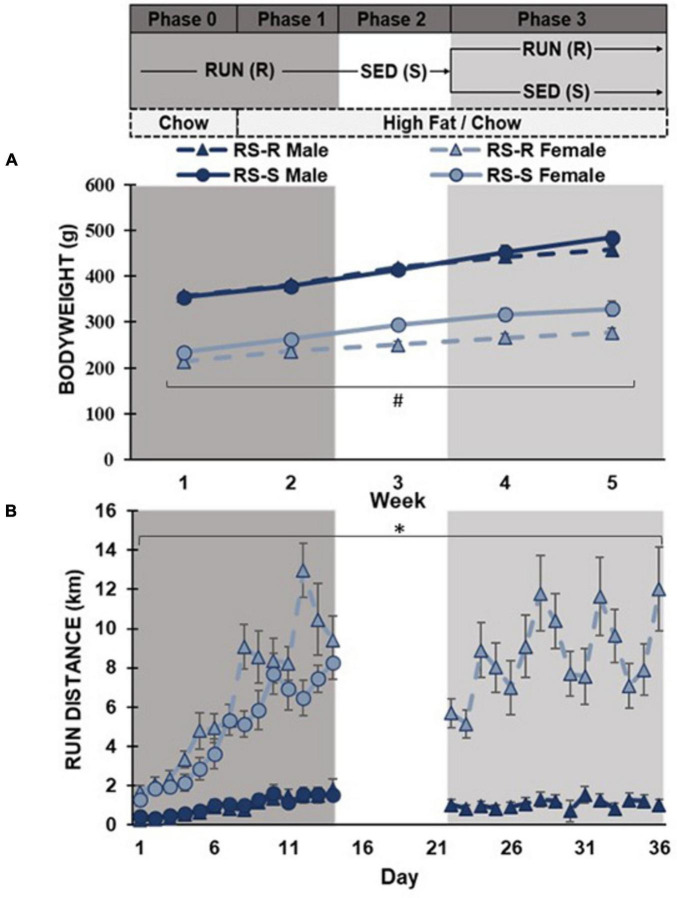
Average daily body weight and running distance with the timeline of experiment listed above the graphs. **(A)** Body weight shown as average daily across each week in grams. RS-S females had significantly larger bodyweights than RS-R females (#*p* < 0.01). **(B)** Average daily running distance in kilometers. Females exhibit higher running distance compared to males (*p < 0.001).

RS-S and RS-R males displayed no significant difference in average daily body weight at week 1 (F_1,15_ 0.2, *p* > 0.05), while RS-S and RS-R females were different (F_1,15_ 9.1, *p* = 0.01). The males and females were run in multiple cohorts, and cohorts were matched for age, which led to minor weight difference between treatment groups in females. A repeated measures ANOVA revealed that this difference within females continued throughout the remainder of the experiment where females the RS-S group weighed more than the RS-R group (F_1,14_ 9.7, *p* = 0.008), yet the difference between the groups increased from 20 g in the first week to 57 g in the fifth week. Males in the RS-S and RS-R groups did not have significantly different bodyweights throughout the 4-week diet preference assessment (F_1,14_ 0.158, *p* > 0.05), but there was a trending difference for RS-S increasing body weight compared to RS-R during the final week (Figure 1).

#### 3.1.2 Running distance

A one-way ANOVA revealed that average daily running distance was higher in females than in males across all four weeks (*p* < 0.001). When comparing RS-R and RS-S average daily running distance in males during the first phase, no difference was found in week 1 (F_1,14_ 0.4, *p* > 0.05) or week 2 (F_1,14_ 0.0, *p* > 0.05). In females, no difference was found in average daily running distance between RS-R and RS-S groups during week 1 (F_1,15_ 2.2, *p* > 0.05), but there was a significant difference during week 2 (F_1,15_ 37.7, *p* < 0.001) (Figure 1).

### 3.2 Dietary preference assessment

#### 3.2.1 Standard chow diet consumption

**FIGURE 2:**
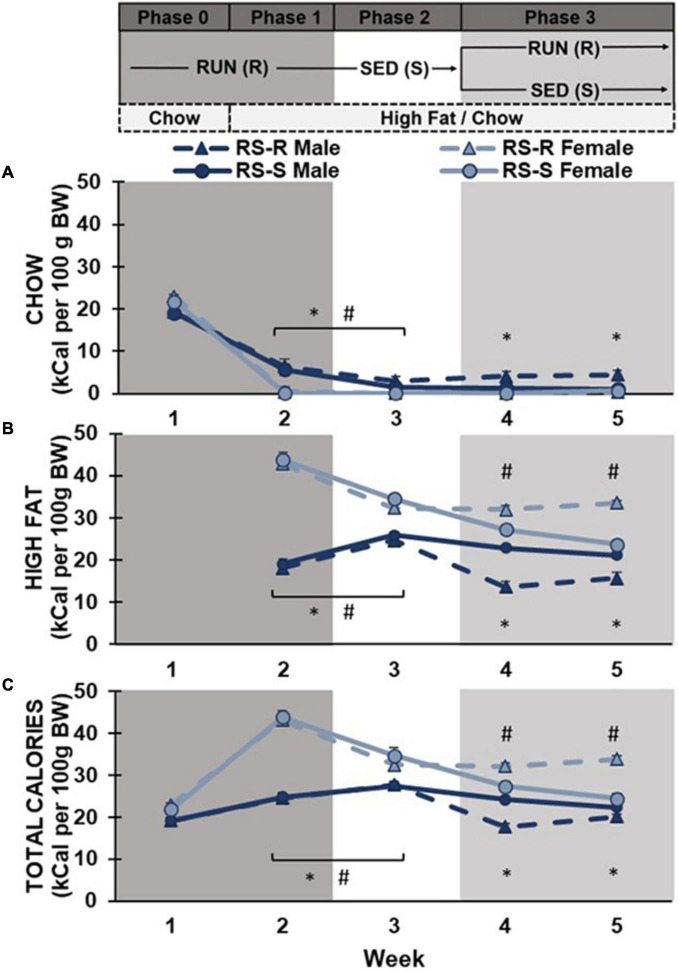
Average daily caloric consumption of chow diet, high fat diet, and total calories normalized to 100 g body weight. **(A)** Chow diet: Both males (*p < 0.05) and females (#*p* < 0.05) showed a significant decrease in chow consumption when transitioned from phase 1 to 2. During phase 3, RS-R males consumed more chow than RS-S males (*p < 0.05). **(B)** High fat diet: Males (*p < 0.05) increased while females (#*p* < 0.05) decreased high fat consumption when transitioned from phase 1 to 2. During phase 3, RS-S males consumed more high fat than RS-R males (*p < 0.05), while RS-R females consumed more high fat than RS-S females (#*p* < 0.05). **(C)** Total caloric consumption: Males (*p < 0.05) increased while females (#*p* < 0.05) decreased calorie consumption when transitioned from phase 1 to 2. During phase 3, RS-S males consumed more calories than RS-R males (*p < 0.05), while RS-R females consumed more calories than RS-S females (#*p* < 0.05).

Phase 0 and 1: A two-way repeated measures ANOVA of chow consumption in Phase 0 and 1 revealed a main effect of week (F_1,28_ 790.6, *p* < 0.001), but no main effect of sex (F_1,28_ 3.9, *p* > 0.05) or group (F_1,28_ 0.7, *p* > 0.05). There was a week by sex interaction (F_1,28_ 51.6, *p* < 0.001), but no week by group (F_1,28_ 0.0, *p* > 0.05), sex by group (F_1,28_ 0.0, *p* > 0.05), or week by group by sex interaction (F_1,28_ 0.4, *p* > 0.05). *Post hoc* comparison showed all rats significantly decreased their chow consumption from week 1 to week 2 (when high fat diet was introduced). There was no difference between males and females chow consumption in week 1 (*p* > 0.05) when only chow was available. When high fat was also available in week 2, males continued to consume small amounts of chow diet, while females stopped consuming chow diet almost entirely (*p* < 0.001) (Figure 2).

Phase 1 and 2: When comparing differences between Phase 1 (RUN condition) and Phase 2 (SED condition), a two-way repeated measures ANOVA on chow consumption revealed a main effect of physical activity (F_1,28_ 21.6, *p* < 0.001) and sex (F_1,28_ 27.9, *p* < 0.001), but not group (F_1,28_ 0.7, *p* > 0.05). There was a physical activity by sex interaction (F_1,28_ 18.5, *p* < 0.001), but no interaction of physical activity by group (F_1,28_ 0.05, *p* > 0.05), sex by group (F_1,28_ 0.4, *p* > 0.05), or physical activity by group by sex (F_1,28_ 0.4, *p* > 0.05). *Post hoc* comparisons showed chow consumption in the RUN condition was significantly higher than the SED condition in both males (*p* < 0.001) and females (*p* = 0.012). Males consumed more chow than females in both physical activity conditions (*p* < 0.001), however, the difference was smaller during the SED condition (Figure 2).

**FIGURE 3:**
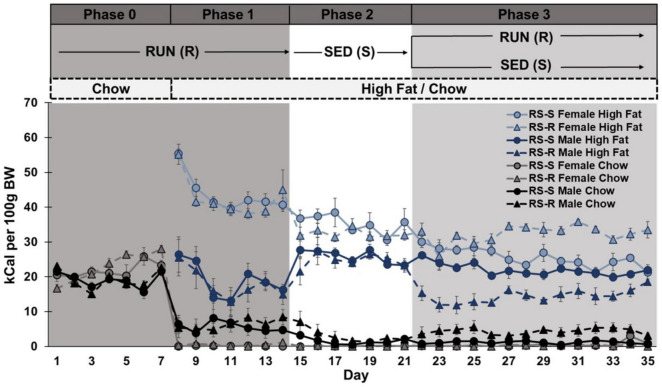
Daily caloric consumption of chow and high fat diet normalized to 100 g body weight.

When comparing chow consumption on Days 14 and 15 (when rats transitioned from RUN to SED condition), a two-way repeated measures ANOVA revealed a main effect of sex (F_1,28_ 17.1, *p* < 0.001), but not day (F_1,28_ 3.3, *p* > 0.05) or group (F_1,28_ 2.1, *p* > 0.05). There were no significant interactions. Regardless of group, females showed a significant decrease in chow consumption from Day 14 to 15 (*p* < 0.001), but males showed no difference (*p* > 0.05) (Figure 3).

Phase 2 and 3: When comparing differences between Phase 2 (SED condition) and Phase 3 week 4 (half remained in SED condition and half transitioned to RUN condition), a two-way repeated measures ANOVA revealed a main effect of sex (F_1,28_ 17.0, *p* < 0.001), but not week (F_1,28_ 3.4, *p* > 0.05) or group (F_1,28_ 3.4, *p* > 0.05). There was an interaction of week by group (F_1,28_ 8.8, *p* = 0.006) and week by group by sex (F_1,28_ 9.1, *p* = 0.005), but not week by sex (F_1,28_ 3.5, *p* > 0.05) or sex by group (F_1,28_ 3.7, *p* > 0.05). *Post hoc* comparison revealed that males consumed more chow than females across both weeks (*p* < 0.001). RS-S males who remained in the SED condition showed no difference between weeks 3 and 4 chow consumption (*p* > 0.05), but RS-R males who were placed into the RUN condition in Phase 3 increased their chow consumption from weeks 3 to 4 (*p* = 0.014). Females showed no difference in chow consumption between weeks 3 to 4, regardless of group (*p* > 0.05) (Figure 2).

When comparing chow consumption on Days 21 and 22 in only RS-R rats (where RS-R rats transitioned from SED to RUN condition), a one-way repeated measures ANOVA revealed a main effect of sex (F_1,14_ 8.3, *p* = 0.012), but not day (F_1,14_ 0.2, *p* > 0.05). There was no interaction of day by sex (F_1,14_ 0.2, *p* > 0.05). Males consumed more chow than females across Days 21 and 22 for both RS-R and RS-S groups (*p* < 0.05). No differences were found between Days 21 and 22 for RS-S male or female rats, who remained in the SED condition (*p* > 0.05) (Figure 3).

Phase 3: A two-way repeated measures ANOVA of chow consumption revealed no main effect of week (F_1,28_ 3.1, *p* > 0.05) in Phase 3. There was a main effect of sex (F_1,28_ 17.7, *p* < 0.001) and group (F_1,28_ 6.3, *p* = 0.018), as well as an interaction of sex by group (F_1,28_ 8.6, *p* = 0.007). There was no interaction of week by group (F_1,28_ 0.2, *p* > 0.05), week by sex (F_1,28_ 0.6, *p* > 0.05), or week by group by sex (F_1,28_ 2.5, *p* > 0.05). *Post hoc* comparisons revealed that males consumed more chow diet than females across Phase 3 (*p* < 0.001). Males in the RUN condition ate significantly more chow than males in the SED condition (F_1,14_ 7.6, *p* = 0.016). There was no difference in chow consumption between RUN and SED condition females (F_1,14_ 1.8, *p* > 0.05). No significant differences were found across weeks when comparing week 4 and 5’s average daily chow consumption (Figure 2).

**FIGURE 4:**
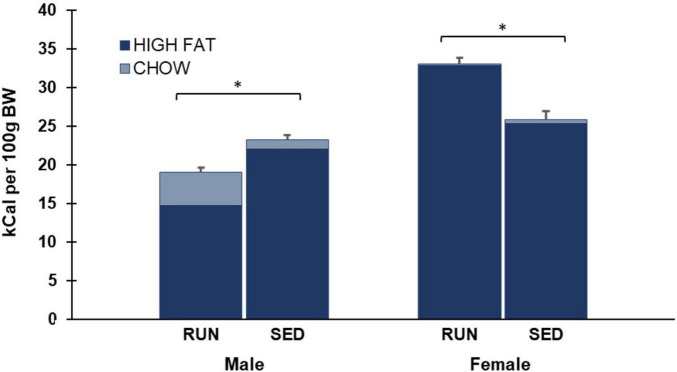
Phase 3 (weeks 4 and 5) average daily caloric consumption of each diet normalized to 100 g body weight. Males who were in the SED condition for the last two weeks consumed more total calories than males who were in the RUN condition. In contrast, females who were in the RUN condition for the last two weeks consumed more total calories than females who were in the SED condition (*p < 0.05).

**FIGURE 5:**
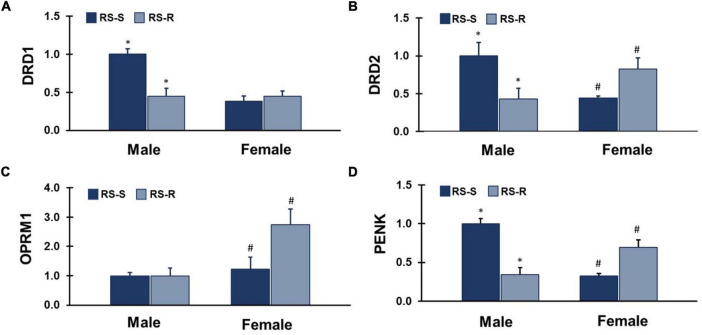
mRNA expression levels of 4 different feeding and reward related genes in the ventral striatum. Values represent a normalization to the male RS-S treatment group. **(A)** DRD1: RS-S males showed increased levels of dopamine receptor type 1 (DRD1) expression compared to RS-R males (*p < 0.05). **(B)** DRD2: RS-S males showed increased levels of dopamine receptor type 2 (DRD2) expression compared to RS-R males (*p < 0.05), and RS-R females showed increased levels of DRD2 expression compared to RS-S females (#*p* < 0.05). **(C)** OPRM1: RS-R females showed increased levels of μ-opioid receptor (OPRM1) expression compared to RS-S females (#*p* < 0.05). **(D)** PENK: RS-S males showed increased levels of proenkephalin (PENK) expression compared to RS-R males (*p < 0.05), and RS-R females showed increased levels of PENK expression compared to RS-S females (#*p* < 0.05).

As shown in Figure 5, data was also analyzed by collapsing intake across both Weeks 4 and 5 of phase 3. A two-way ANOVA of average daily chow consumption in phase 3 revealed a main effect of sex (F_1,28_ 17.5, *p* < 0.001), group (F_1,28_ 6.4, *p* = 0.017), and a significant interaction of sex by group (F_1,28_ 8.5, *p* = 0.007) (Figure 4).

#### 3.2.2 High-fat diet consumption

Phases 1 and 2: When comparing average daily high fat consumption differences between Phase 1 (RUN condition) and Phase 2 (SED condition), a two-way repeated measures ANOVA revealed no main effect of physical activity (F_1,28_ 2.3, *p* > 0.05), or group (F_1,28_ 1.1, *p* > 0.05). There was a main effect of sex (F_1,28_ 160.2, *p* < 0.001), and a significant physical activity by sex interaction (F_1,28_ 66.8, *p* < 0.001). There was no interaction of physical activity by group (F_1,28_ 0.1, *p* > 0.05), sex by group (F_1,28_ 0.0, *p* > 0.05), or physical activity by sex by group (F_1,28_ 0.1, *p* > 0.05). *Post hoc* comparisons revealed no differences in high fat consumption found between RS-S and RS-R groups during Phases 1 and 2 in both males (*p* > 0.05) and females (*p* > 0.05) when both groups were undergoing the same treatment. Male rats showed a significant increase in high fat diet consumption when transitioned from RUN condition to SED condition (*p* < 0.001). In contrast, females showed a significant decrease in high fat consumption when transitioned from the RUN condition to the SED condition (*p* < 0.001) (Figure 2).

When comparing high fat consumption on Days 14 and 15 (where rats transitioned from RUN to SED condition), a two-way repeated measures ANOVA revealed a main effect of sex (F_1,28_ 75.8, *p* < 0.001), but not day (F_1,28_ 0.01, *p* > 0.05) or group (F_1,28_ 0.9, *p* > 0.05). There was a significant interaction of day by sex (F_1,28_ 8.4, *p* < 0.001), but not day by group (F_1,28_ 1.3, *p* > 0.05), sex by group (F_1,28_ 0.7, *p* > 0.05), or day by sex by group (F_1,28_ 0.1, *p* > 0.05). Regardless of group, males showed a significant increase in high fat consumption from Day 14 to 15 (*p* < 0.001), but no significant change in females (*p* > 0.05) (Figure 3).

Phase 2 and 3: When comparing high fat consumption differences between Phase 2 (SED condition) and Phase 3 week 4 (half remain in SED condition and half transition to RUN condition), a two-way repeated measures ANOVA revealed a main effect of week (F_1,28_ 52.7, *p* < 0.001), sex (F_1,28_ 94.1, *p* < 0.001), and group (F_1,28_ 4.6, *p* = 0.042). There was a significant interaction of sex by group (F_1,28_ 9.9, *p* = 0.004), week by group by sex (F_1,28_ 21.9, *p* < 0.001), but not week by sex (F_1,28_ 3.6, *p* > 0.05) or week by group (F_1,28_ 0.3, *p* > 0.05). *Post hoc* comparison revealed that females consumed more high fat than males across both weeks (*p* < 0.001). While both RS-S males who remained in the SED condition (*p* = 0.04) and RS-R males who transitioned to RUN condition (*p* < 0.001) decreased their high fat consumption from Phase 2 to 3, RS-R condition males decreased at a much larger scale (*p* < 0.001). RS-S females who remained in the SED condition decreased their high fat consumption from Phase 2 to 3 (*p* = 0.010), while high fat consumption for RS-R females who were placed in RUN condition in Phase 3 did not differ between phases (*p* > 0.05) (Figure 2).

When comparing high fat consumption levels between Days 21 and 22 in only RS-R rats (where RS-R rats transitioned from SED to RUN condition), a one-way repeated measures ANOVA revealed a main effect sex (F_1,14_ 805.0, *p* < 0.001), but not day (F_1,14_ 3.0, *p* > 0.05). There was an interaction of day by sex (F_1,14_ 5.2, *p* < 0.05). RS-R males significantly decreased their high fat consumption from Days 21 to 22 (*p* < 0.05), but high fat consumption in RS-R female was unchanged (*p* > 0.05). Females consumed more high fat than males across Days 21 and 22 in both RS-R and RS-S rats (*p* < 0.001). No differences were found between Days 21 and 22 for RS-S male or female rats, who remained in the SED condition (*p* > 0.05) (Figure 3).

Phase 3: A two-way repeated measures ANOVA revealed a main effect of sex, (F_1,28_ 112.4, *p* < 0.001), but no main effect of week (F_1,28_ 0.9, *p* > 0.05) or group (F_1,28_ 0.1, *p* > 0.05). There was a significant interaction of week by group (F_1,28_ 28.8, *p* < 0.001), and sex by group (F_1,28_ 51.1, *p* < 0.001), but not for week by sex (F_1,28_ 1.0, *p* > 0.05) or week by sex by group (F_1,28_ 1.3, *p* > 0.05). *Post hoc* comparisons revealed that females consumed more high fat than males across both weeks 4 and 5 (*p* < 0.001). Males who were reintroduced to the RUN condition consumed less high fat when compared to the males in the SED condition (F_1,14_ 23.8, *p* < 0.001). In contrast, females who were reintroduced to the RUN condition significantly increased their high fat consumption, compared to their SED counterparts (F_1,14_ 28.4, *p* < 0.001). When comparing high fat consumption between week 4 and 5, rats in the RUN condition increased their high fat consumption (*p* < 0.001), while rats in the SED condition decreased their high fat consumption over time (*p* = 0.013) (Figure 2).

As shown in Figure 5, data was also analyzed by collapsing intake across both Weeks 4 and 5 of phase 3. A two-way ANOVA of average daily high fat consumed in phase 3 revealed a main effect of sex (F_1,28_ 112.4, *p* < 0.001) and a significant interaction of sex by group (F_1,28_ 51.1, *p* < 0.001), but no main effect of group (F_1,28_ 0.1, *p* > 0.05) (Figure 4).

#### 3.2.3 Total diet consumption

Phase 1 and 2: A two-way repeated measures ANOVA of total calories consumed across Phase 1 (RUN condition) and Phase 2 (SED condition) revealed a main effect of physical activity (F_1,28_ 19.1, *p* < 0.001) and sex (F_1,28_ 190.8, *p* < 0.001), but not group (F_1,28_ 0.6, *p* > 0.05). There was an interaction of physical activity by sex (F_1,28_ 64.3, *p* < 0.001), but no interaction of physical activity by group (F_1,28_ 0.1, *p* > 0.05), sex by group (F_1,28_ 0.6, *p* > 0.05), or physical activity by sex by group (F_1,28_ 0.4, *p* > 0.05). *Post hoc* comparisons showed that females had significantly higher caloric consumption than males (*p* < 0.001). Male rats showed a significant increase in caloric consumption when transitioned from RUN condition to SED condition (*p* = 0.018). In contrast, females showed a significant decrease in caloric consumption when transitioned from the RUN condition to the SED condition (*p* < 0.001) (Figure 2).

When comparing total consumption on Days 14 and 15 (where rats transitioned from RUN to SED condition), a two-way repeated measures ANOVA revealed a main effect sex (F_1,28_ 79.2, *p* < 0.001), but not day (F_1,28_ 0.05, *p* > 0.05) or group (F_1,28_ 0.00, *p* > 0.05). There was an interaction of day by sex (F_1,28_ 7.4, *p* = 0.01), but not day by group (F_1,28_ 1.4, *p* > 0.05), sex by group (F_1,28_ 0.00, *p* > 0.05), or day by sex by group (F_1,28_ 0.2, *p* > 0.05). Regardless of group, males showed a significant increase in total consumption from Day 14 to 15 (*p* < 0.001), but female’s decrease in total consumption from Day 14 to 15 was not significant (*p* > 0.05) (Figure 3).

Phase 2 and 3: When comparing differences between Phase 2 (SED condition) and Phase 3 week 4 (half remain in SED condition and half transition to RUN condition), a two-way repeated measures ANOVA revealed a main effect of week (F_1,28_ 46.9, *p* < 0.001), sex (F_1,28_ 90.0, *p* < 0.001), but not group (F_1,28_ 1.4, *p* > 0.05). There was an interaction of sex by group (F_1,28_ 7.9, *p* = 0.009) and week by group by sex (F_1,28_ 20.2, *p* < 0.001), but not week by sex (F_1,28_ 3.5, *p* > 0.05) or week by group (F_1,28_ 0.0, *p* > 0.05). *Post hoc* comparison revealed that females consumed more calories than males across both weeks (*p* < 0.001). While both RS-S males who remained in the SED condition (*p* = 0.011) and RS-R males who were transitioned to RUN condition (*p* < 0.001) decreased their total caloric consumption from weeks 3 to 4, RS-R males decreased to a greater extent (*p* < 0.001). RS-S females who remained in the SED conditioned decreased their total caloric consumption from weeks 3 to 4 (*p* = 0.010), while total caloric consumption for RS-R females who were transitioned to RUN condition did not differ between weeks 3 and 4 (*p* > 0.05) (Figure 2).

When comparing total consumption between Day 21 and 22 in only RS-R rats (where RS-R rats transition from SED to RUN condition), a one-way repeated measures ANOVA revealed a main effect of day (F_1,14_ 5.6, *p* = 0.05) and sex (F_1,14_ 1013.4, *p* < 0.001), and a significant interaction of day by sex (F_1,14_ 9.2, *p* < 0.01). RS-R males decreased their total consumption from Day 21 to 22 (*p* < 0.05), but there was no difference in RS-R female high fat consumption (*p* > 0.05). Females consumed more calories than males across Day 21 and 22 in both RS-R and RS-S rats (*p* < 0.001). No differences were found between Days 21 and 22 for RS-S male or female rats, who remained in the SED condition (*p* > 0.05) (Figure 3).

Phase 3: A two-way repeated measures ANOVA of total calories consumed revealed a main effect of sex (F_1,28_ 115.3, *p* < 0.001), but no main effect of week (F_1,28_ 0.3, *p* > 0.05) or group (F_1,28_ 3.2, *p* > 0.05). There was a significant interaction of week by group (F_1,28_ 37.3, *p* < 0.001), and group by sex (F_1,28_ 53.5, *p* < 0.001), but no interaction of week by sex (F_1,28_ 1.6, *p* > 0.05), or week by sex by group (F_1,28_ 0.04, *p* > 0.05). *Post hoc* comparisons showed that females had significantly higher caloric consumption than males across Phase 3 (*p* < 0.001). Males who were reintroduced to the RUN condition had lower total caloric consumption compared to the males in the SED condition (F_1,14_ 19.3, *p* < 0.001). In contrast, females who were reintroduced to the RUN condition had higher caloric consumption when compared to their counterparts in the SED condition (F_1,14_ 34.2, *p* < 0.001). Both male and female rats in the RUN condition increased their caloric consumption from Week 4 to 5 (*p* < 0.001), and all rats in the SED condition decreased their caloric consumption from week 4 to 5 (*p* < 0.001) (Figure 2).

As shown in Figure 5, data was also analyzed by collapsing intake across both Weeks 4 and 5 of phase 3. A two-way ANOVA of average daily total calories consumed in phase 3 revealed a main effect of sex (F_1,28_ 115.1, *p* < 0.001) and a significant interaction of sex by group (F_1,28_ 53.9, *p* < 0.001), but no main effect of group (F_1,28_ 4.0, *p* > 0.05) (Figure 4).

### 3.3 Correlation between running distance and consumption

A Pearson’s correlation coefficient was computed to assess the linear relationship between wheel running distance and intake of either of the 2 diets in RS-R males, RS-S males, RS-R females, and RS-S females separately. No significant correlation was found for wheel running distance and high fat for any of the phases (*p* > 0.05). In addition, no correlation was found for wheel running distance and chow consumption for any phase (*p* > 0.05). Tables 1, 2 show Pearson’s correlation coefficients for all phases for both chow and high fat diets.

**TABLE 1:** Pearson’s correlation coefficient of average daily running distance and average daily high fat consumption for Phase 1 and Phase 3.

	Phase 1	Phase 3
RS-R Males	*r*(6) = 0.119, *p* > 0.05	*r*(6) = −0.235, *p* > 0.05
RS-S Males	*r*(6) = 0.213, *p* > 0.05	
RS-R Females	*r*(6) = 0.008, *p* > 0.05	*r*(6) = 0.200, *p* > 0.05
RS-S Females	*r*(6) = 0.525, *p* > 0.05	

**TABLE 2:** Pearson’s correlation coefficient of average daily running distance and average daily chow consumption for Phase 0, Phase 1, and Phase 3.

	Phase 0	Phase 1	Phase 3
RS-R Males	*r*(6) = 0.643, *p* > 0.05	*r*(6) = 0.027, *p* > 0.05	*r*(6) = 0.525, *p* > 0.05
RS-S Males	*r*(6) = 0.453, *p* > 0.05	*r*(6) = −0.234, *p* > 0.05	
RS-R Females	*r*(6) = 0.157, *p* > 0.05	*r*(6) = 0.034, *p* > 0.05	*r*(6) = −0.259, *p* > 0.05
RS-S Females	*r*(6) = −0.542, *p* > 0.05	*r*(6) = 0.449, *p* > 0.05	

### 3.4 Ventral striatum mRNA expression

#### 3.4.1 DRD1 mRNA expression

A two-way ANOVA for DRD1 mRNA expression revealed a main effect of group (F_1,17_ 26.9, *p* < 0.001), and sex (F_1,17_ 21.5, *p* < 0.001). There was an interaction of group by sex (F_1,17_ 59.2, *p* < 0.001) (Figure 5). RS-S males who were in the SED condition during the last 2 weeks expressed greater levels of DRD1 than RS-R males who were in the RUN condition during the last 2 weeks (F_1,12_ 17.0, *p* < 0.001). There was no difference between the RS-R condition and RS-S females (F_1,12_ 0.5, *p* > 0.05).

#### 3.4.2 DRD2 mRNA expression

A two-way ANOVA for DRD2 mRNA expression revealed no main effect of group (F_1,17_ 0.2, *p* > 0.05), or sex (F_1,17_ 0.1, *p* > 0.05). There was an interaction of group by sex (F_1,17_ 10.7, *p* = 0.004) (Figure 5). RS-S Males who were in the SED condition during the last 2 weeks expressed greater levels of DRD2 than RS-R males who were in the RUN condition during the last 2 weeks (F_1,13_ 6.0, *p* = 0.029). In contrast, RS-S females who were in the SED condition during the last 2 weeks expressed lower levels of DRD2 than RS-R females who were in the RUN condition during the last 2 weeks (F_1,11_ 5.8, *p* = 0.035).

#### 3.4.3 OPRM1 mRNA expression

A two-way ANOVA for OPRM1 mRNA expression revealed a main effect of sex (F_1,17_ 6.6, *p* = 0.020), but not group (F_1,17_ 1.4, *p* > 0.05). There was no interaction of group by sex (F_1,17_ 2.6, *p* > 0.05) (Figure 5). Females showed greater levels of OPRM1 than males when groups were collapsed. RS-S females who were in the SED condition during the last 2 weeks expressed lower levels of OPRM1 than RS-R females who were in the RUN condition during the last 2 weeks (F_1,13_ 5.0, *p* = 0.043). There was no difference between RS-R and RS-S males (F_1,11_ 0.0, *p* > 0.05).

#### 3.4.4 PENK mRNA expression

A two-way ANOVA for PENK mRNA expression revealed a main effect of group (F_1,17_ 6.1, *p* = 0.024), but not sex (F_1,17_ 3.7, *p* > 0.05). There was an interaction of group by sex (F_1,17_ 44.4, *p* < 0.001) (Figure 5). RS-S males who were in the SED condition during the last 2 weeks expressed greater levels of PENK than RS-R males who were in the RUN condition during the last 2 weeks (F_1,12_ 33.3, *p* < 0.001). In contrast, RS-S females who were in the SED condition during the last 2 weeks expressed lower levels of PENK than RS-R females who were in the RUN condition during the last 2 weeks (F_1,13_ 12.4, *p* = 0.004).

## 4 Discussion

**FIGURE 6:**
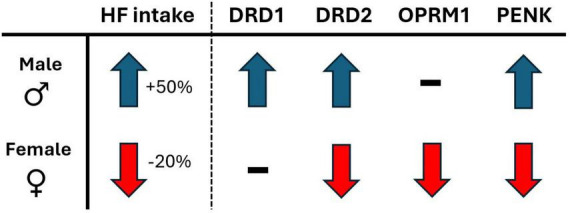
The sex-dependent directional shift in behavioral and mRNA measures in response to sedentary (locked wheel) conditions, compared to voluntary wheel running access conditions. Behavioral measures: HF, high-fat diet intake; gene expression (mRNA) measures; DRD1, dopamine receptor type 1; DRD2, dopamine receptor type 2; OPRM1, μ-opioid receptor 1; PENK, proenkephalin.

The present study explored the effects of voluntary physical activity levels on a 2-diet preference test between palatable high fat diet and a less palatable, nutritionally balanced chow diet, in both male and female rats. The major findings of the study reveal that voluntary wheel running access, compared to sedentary locked wheel, shifts preference and consumption of palatable diet in the opposite direction for males and females. Indeed, males consumed 50% *more* high fat while in SED, compared to RUN condition. In contrast, females consumed 20% *less* high fat while in SED, compared to RUN condition (for summary, see Figure 6). The response in females would appear to be adaptive, as they decrease palatable food intake when in sedentary home cage conditions. In contrast, the opposite response observed in males would appear to be a potential maladaptive response, overconsuming palatable food when in sedentary home cage conditions.

To further understand the relationship between physical activity and palatability-driven feeding, the present study also followed a reversal/ABA research design, providing additional experimental control over the interaction and increased internal validity to overall findings. The results demonstrate that the sex-dependent interaction between physical activity and diet preference and consumption patterns respond quickly to changing physical activity conditions, reversing within the first 24 h of alternating between RUN and SED conditions. Lastly, these sex dependent feeding behaviors were associated with parallel sex-dependent changes to critical reward-related gene expression patterns in the Acb (for summary, see Figure 6), suggesting a role for both opioid and dopamine as potential regulatory processes underlying these behaviors.

One potential explanation for the sex dependent effect of voluntary wheel running in the present study could be the influence of metabolic changes due to the discrepancy in running behavior between males and females (Tokuyama et al., 1982; Stiegler and Cunliffe, 2006). However, there was no correlation in either males or females between running distance and intake levels of either diet at any phase of the experiment. This lack of correlation aligns with previous findings from our lab and others showing that while rats exhibit a range of daily running distances, no correlation between running distance and palatable diet intake has been observed (Shapiro et al., 2011; Scarpace et al., 2012; Lee et al., 2017, 2019a). This suggests that the critical factor underlying the influence of the RUN condition on feeding behavior is access to the voluntary running wheel, not the distance ran.

Another potential explanation for the sex-dependent interaction of physical activity and feeding patterns could be an influence of sex-specific hormones, a question most often explored in females. It is well known that female rats exhibit markedly higher running distances compared to males, and that this difference is estrogen dependent (Mathis et al., 2024). While the current study didn’t directly address the role of sex hormones, it did reveal that there was no correlation between high fat consumption and running distance, in either females or males. Also, it has been shown that ovariectomized female rats, compared to intact rats, display similar palatable feeding behavior in response to physical activity (Moody et al., 2015). While these findings suggest that metabolic or hormonal mechanisms may not be the key mediators underlying the sex-dependent effects in the present study, additional approaches to investigating either direct or indirect roles of both male and female sex hormones are certainly warranted (McCarthy et al., 2012; Park et al., 2016).

The mesolimbic reward pathway has revealed many sex-dependent physiological differences for both drug and natural rewards (Contet et al., 2004; Avena et al., 2008; Le Merrer et al., 2009; Bobzean et al., 2014; Harp et al., 2020; Orsini et al., 2022), yet this pathway remains relatively unexplored for mediating the sex-dependent interaction of physical activity and feeding behavior. The processes underlying the rewarding properties of both palatability-driven feeding and voluntary wheel running have been well documented (Garland et al., 2011; Novak et al., 2012), as both palatable diet consumption and voluntary wheel running have been shown to be altered through systemic (Cooper, 1980; Marks-Kaufman, 1982; Gosnell et al., 1990; Sisti and Lewis, 2001) and direct opioid administration into the ventral striatum (Zhang et al., 1998; Will et al., 2006, 2007; Ruegsegger et al., 2015). Considering this overlap, the opioid system is a likely candidate for mediating the interaction of physical activity and feeding behavior. Long-term voluntary wheel running leads to adaptations in the gene expression patterns associated with reward in the Acb (Werme et al., 2002; Greenwood et al., 2011). Previous studies, conducted mostly in males, have shown that access to voluntary wheel running causes animals to be less sensitive to the reinforcing properties of drugs of abuse (Mustroph et al., 2011; Lynch et al., 2013), as voluntary wheel running decreased acquisition and self-administration response rates for cocaine, heroin, methamphetamine, and nicotine (Lacy et al., 2014; Bardo and Compton, 2015; Sanchez et al., 2015).

Previous findings in our lab revealed a sex dependent effect of voluntary wheel running access on a 3-diet preference test, where RUN females increased their high-fat diet intake compared to SED females, but RUN males decreased high-fat intake in favor of a high-sucrose diet compared to SED males (Lee et al., 2017). In the same study, females in the RUN condition, compared to SED condition, had increased OPRM1 and PENK mRNA expression in the Acb, but no significant differences in gene expression were found in males. The lack of mRNA expression differences between RUN and SED conditions in males might have been due to multiple palatable diets being available. While RUN males did reduce fat intake (as in the present study), they also significantly increased sucrose intake, when compared to SED males (Lee et al., 2017). Therefore, the present study sought to simplify the diet preference test, providing access to only one palatable diet and standard chow, which allowed for easier interpretation of both behavioral and mRNA expression changes. Interestingly, the DRD2 and PENK mRNA expression levels track the high-fat diet consumption for both sexes, with decreased expression in RUN males and increased expression in RUN females. DRD1 and OPRM1 gene expression levels tracked with high fat diet consumption for males and females, respectively. These mRNA expression differences observed in response to RUN vs. SED conditions provide support for these ventral striatum reward signaling processes being likely mediators of the sex-dependent interaction between physical activity and feeding patterns. Future studies will be necessary to isolate the independent influence of physical activity and the resulting effects on these gene expression patterns.

Another potential theory to explain these sex dependent results involves the reward substitution principle; a process involving two or more rewarding events reducing the appetitive nature of each other (Lynch et al., 2013; Bardo and Compton, 2015). Previously, it has been shown that female rats demonstrate higher levels for the self-administration of drugs of abuse, palatable food intake, and voluntary wheel running, when compared to males (Mathes and Kanarek, 2001; Lett et al., 2002; Carroll et al., 2004; Carroll and Anker, 2010; Smith and Pitts, 2012; Lee et al., 2017). Therefore, the ceiling for the behavioral expression related to the rewarding properties of high-fat diet or voluntary wheel running would be higher for females, compared to males. While males decreased their high fat consumption when running wheel was available, the opposite occurred in females. This aligns with reward substitution, as male’s reward “ceiling” may lead to greater reward competition between running and high fat diet when concurrently accessible, while females may experience a reduced level of competition. It also raises the importance of methodological approach used to assess feeding behavior (i.e., novel feeding chambers or home cage), where feeding is typically assessed with or without concurrent access to the running wheel, respectively, which can produce different outcomes in feeding behavior (Lee et al., 2017, 2019b).

Previous research has found that chronic access to either palatable diets or voluntary wheel running produces similar neuronal plasticity in the Acb and associated reward pathways (Kelley et al., 2002; Vargas-Pérez et al., 2003; Greenwood et al., 2011). Considering the current study only assessed mRNA gene expression, it would be interesting to use a different experimental approach to investigate the directional influence occurring between physical activity and the gene expression patterns. One approach is to assess the effect of intra-Acb administration of the selective μ-opioid receptor agonist D-Ala2, NMe-Phe4, Glyol5-enkephalin (DAMGO). Intra-Acb DAMGO administration has been well characterized to increase consumption of palatable diets by increasing the rewarding and palatable properties of preferred food, rather than increasing the state of “hunger” or negative energy balance (Peciña and Berridge, 2000; Kelley et al., 2002; Hanlon et al., 2004; Will et al., 2009). Previous research from our lab demonstrated that in females, intra-Acb DAMGO feeding behavior of palatable diets was altered by access to a running wheel, where females in the RUN condition responded to lower doses of DAMGO than SED condition females. In male rats, there was no effect of RUN condition compared to SED condition on DAMGO increased palatable diet consumption (Lee et al., 2019b). However, this study tested intra-Acb DAMGO feeding behaviors in a separate feeding chamber, with no access to a running wheel during the feeding test. As the present study’s results suggest, access to running wheel in males may cause reward competition between the running wheel and high fat diet. Future studies examining the impact of access to a running wheel being present during DAMGO feeding test will help further characterize the interaction of physical activity and feeding behavior.

As mentioned previously, the present study also examined if RUN effects are sensitive to reversal by SED condition in male and female rats. The results demonstrated in general these effects were indeed reversible. Male rats show an immediate (24 h) increase in high fat consumption when transitioned from RUN to SED condition, and then a subsequent decrease in high fat consumption when transitioned back from SED to RUN, with the changes persisting throughout the final two weeks. Within females, transition effects of physical activity conditions were also observed, as they initially showed a decrease in high fat when transitioned from RUN to SED, yet slowly increased high fat intake after they transitioned from SED back to RUN condition, compared to the rats that remained in SED condition. These findings all lend support that run effects on palatable diet consumption are reversible in both males and females.

Overall, the present data strongly suggest that physical activity has a sex-dependent effect on palatable diet preference and feeding behavior. Furthermore, in line with the dissociative sex-dependent influence of physical activity condition on feeding behavior, ventral striatal opioid- and dopamine-related mRNA expression also were found to parallel these behavioral effects. The sex-dependent effects of physical activity were also shown to reverse in response to alternating physical activity conditions, suggesting the underlying opioid and dopaminergic mediators may also respond similarly. Considering the widespread prevalence of sedentary behavior and excessive consumption of energy-rich, palatable diets, it seems critical to further our understanding of how a sedentary lifestyle impacts males and females differently. Continued research into this interaction should also provide insight into whether the sex-dependent influence of physical activity on palatable hedonic feeding might also extend to drugs of abuse and in turn, lead to sex-specific approaches to treatment options.

## Data availability statement

The raw data supporting the conclusions of this article will be made available by the authors, without undue reservation.

## Ethics statement

The animal study was approved by the Animal Care and Use Committee (ACUC) of University of Missouri. The study was conducted in accordance with the local legislation and institutional requirements.

## Author contributions

CK: Conceptualization, Data curation, Formal analysis, Investigation, Methodology, Project administration, Validation, Visualization, Writing – original draft, Writing – review & editing. YC: Investigation, Writing – review & editing. DS: Data curation, Formal analysis, Investigation, Writing – review & editing. TAS: Investigation, Writing – review & editing. EK: Investigation, Writing – review & editing. TH: Investigation, Writing – review & editing. CS: Investigation, Writing – review & editing. TRS: Supervision, Writing – review & editing. VV-P: Writing – review & editing. MW: Conceptualization, Investigation, Methodology, Project administration, Supervision, Validation, Writing – review & editing.
